# Less invasive surfactant administration via infant feeding tube versus InSurE method in preterm infants: a randomized control trial

**DOI:** 10.1038/s41598-022-23557-3

**Published:** 2022-12-19

**Authors:** Rohit Anand, Sushma Nangia, Gunjana Kumar, M. Vishnu Mohan, Ajay Dudeja

**Affiliations:** Department of Neonatology, Lady Hardinge Medical College and Associated Kalawati Saran Children’s Hospital, New Delhi, India 110001

**Keywords:** Diseases, Medical research

## Abstract

There is growing evidence that less invasive surfactant administration (LISA) is a better alternative to the standard Intubate-surfactant-extubate (InSurE) procedure in spontaneously breathing preterm infants with RDS. The infant feeding tube is easily available and cost-effective in comparison to special catheters used for surfactant administration in various studies on LISA and cost-effective health care is the need of the hour for countries like ours which are Low and middle-income countries(LMICs).The present study was planned to compare the total duration of respiratory support in preterm babies between 26 to 34 weeks of gestation with RDS requiring surfactant therapy administered by LISA technique using an infant feeding tube or InSurE method. In this unblinded randomised controlled trial, 150 infants were allocated to LISA (n = 74) or InSurE group (n = 76). An 8F feeding tube was used for surfactant delivery in the LISA group. The primary outcome was the total duration of respiratory support required and secondary outcomes included the proportion of babies developing BPD, IVH, PDA, NEC, ROP, air leaks, CPAP failure, and those requiring a repeat dose of surfactant along with the duration of hospitalization, time to regain birth weight and Death. The baseline variables including birth weight and gestation age were similar in the two groups. Nearly 27% of the mothers did not receive any dose of antenatal steroids (ANS) while around 37% of the mothers received complete course of ANS. A high proportion of babies (57%) were delivered by cesarean section. Intrapharyngeal reflux was significantly more in babies who received surfactant with the LISA method in comparison to InSurE technique (32% v/s 3%, p < 0.001). There was no statistically significant difference in the primary outcome of the total duration of respiratory support in both groups with a median duration of 120 h, 95% CI (69–235), and p = 0.618. The need for invasive mechanical ventilation was significantly lower in the LISA group (p = 0.017) with RR (95% CI) 0.498 (0.259–0.958). The rate of CPAP failure was significantly lower in the LISA group (p = 0.005) with RR (95% CI) 0.55 (0.34–0.89). In this study, the total duration of hospital stay was reduced in the LISA group (19 days) compared to InSurE group (26 days), although the same was not statistically significant. LISA with an 8F feeding tube is feasible and an effective strategy for surfactant administration which resulted in a significant reduction in CPAP failure and the need for invasive mechanical ventilation.

**Trial registration**: www.ctri.nic.in id CTRI/2020/05/025360. Trial was registered at CTRI on 26/05/2020. First case of trial was enrolled on 28/05/2020.

## Introduction

Respiratory distress syndrome (RDS) is a common neonatal condition in preterm infants. Early nasal CPAP and selective administration of surfactant via the endotracheal tube are widely used in the treatment of RDS in preterm infants^1^. There is growing evidence that LISA/MIST is a better alternative to the InSurE procedure in spontaneously breathing preterm infants with RDS. In a recent systematic review, Isayama et al. have described that LISA decreased the need for mechanical ventilation as well as reduced the incidence of intraventricular hemorrhage and bronchopulmonary dysplasia^2^. The infant feeding tube is easily available in all NICUs and is far more cost-effective in comparison to special catheters used previously in various studies on LISA^3^. There is a dearth of studies on LISA from low- and middle-income countries where the availability and cost of special catheters may pose challenges in the implementation of LISA/MIST. The present study was planned to evaluate the effect of administering surfactant by LISA method using an 8F orogastric feeding tube over the traditional InSurE method on the duration of mechanical ventilation and other modalities of respiratory support.

## Material and methods

### Subjects and settings

This unblinded randomized control trial was conducted at a tertiary care neonatal unit in New Delhi, India. The ethical approval for the study was obtained from the Ethics Committee for Human Research, Lady Hardinge Medical College, New Delhi, India. All methods were performed in accordance with the relevant guidelines and regulations. We included hemodynamically stable, intramural preterm infants between 26 to 34 weeks of gestation, who presented with features of RDS within six hours of life and required surfactant therapy on nCPAP (requiring FiO_2_ > 30%) after obtaining parental consent. Infants were excluded if they: 1) had a major congenital anomaly, (2) developed shock prior to randomization, (3) had suffered severe asphyxia (Apgar score < 4 at 5 minutes and cord Ph < 7.0) or (4) were already intubated at the time of first surfactant dose.

### Randomisation and intervention

The infants were randomized into the LISA or InSurE group within six hours of birth using opaque sealed envelopes to ensure allocation concealment. Blinding of the investigators or primary caregivers was not possible due to the nature of the intervention. Gestational age assessment was based on the last menstrual period and an early dating scan, or on the Expanded New Ballard score if the former were unavailable or had a discrepancy of 2 weeks or more.

### Trial treatment

Participants were randomized to one of the following treatment arms:

- Arm A: Less invasive surfactant administration with 8F orogastric feeding tube.
- Arm B: Surfactant administration using InSurE method.

### LISA procedure

The procedure was performed in the NICU by a trained neonatologist and a staff nurse. Before the LISA procedure, the infant was positioned in the sniffing position. Heart rate and SpO2 were monitored throughout the procedure. Direct laryngoscopy was performed and an 8 Fr feeding tube was inserted to the desired depth without the use of Magill forceps or removal of the CPAP interface. The required tip to lip length was calculated as weight in kilograms plus 6 cm as per our prevalent NICU method. Due to technical difficulty in visualizing the exact length of the feeding tube beyond the vocal cord, we used the tip to lip length for LISA.

No sedation or premedication was used but nesting and swaddling were done during the procedure for the comfort of the baby. After feeding tube placement, the laryngoscope was removed. Survanta at the dose of 100 mg/kg was used for surfactant replacement therapy. The surfactant syringe was connected to the catheter’s bulb, and the surfactant was instilled slowly and continuously over 4 minutes. After complete dose administration, the feeding tube was withdrawn. If the first attempt at catheterization of the trachea was not possible in 20 s to 30 s, then the catheter was removed, a repeat attempt was done after recovery, and the maximum attempt limit was kept to three attempts. If the infant continued to require FiO_2_ of more than 30% after 6 h of initial surfactant therapy, a second dose of surfactant was given by the same technique.

### InSurE procedure

In InSurE procedure, the infant was positioned as per standard intubation procedure**.** Laryngoscopy and intubation were done after the removal of the CPAP mask/prongs. The feeding tube was prepared for desired depth as per the selected endotracheal tube before surfactant administration**.** Once the endotracheal tube (ET) position was adjudged to be at the correct position, the syringe filled with the surfactant was attached to a feeding tube which was now inserted into the ET to the desired depth. The total dose of surfactant was instilled in four equal aliquots. Bag and tube ventilation was done in between the aliquots of surfactant. After completion of surfactant administration, the endotracheal tube was immediately removed and the baby was shifted back to CPAP with mask/prongs^4^.

### After surfactant administration

If the patient required another dose of surfactant (FiO_2_ > 0.3, paCO_2_ ≥ 60 mmHg, Pao2 < 50 mmHg) even after 4–6 h of the first dose, the second dose of surfactant was administered, and the same procedure was used as during the first surfactant instillation. Maximum acceptable settings were sustained CPAP pressure of 7 cm H_2_O along with FiO2 0.6, infants exceeding these limits were shifted to non-invasive mechanical ventilation. Indications for intubation were documented respiratory acidosis (pH < 7.2) and apneic episodes requiring PPV or repeated apneic episodes even on non-invasive mechanical ventilation. Criteria for the repeat dose of surfactant and mechanical ventilation in the event of failure of CPAP were the same in both groups. All subjects were followed up till discharge or death.

### Outcome variables and their measurements

All neonates received standard care as per the unit protocol (including kangaroo mother care, use of caffeine, early enteral feeding, and developmentally supportive care). The primary outcome of this study was to compare the total duration of respiratory support between the two methods used for surfactant administration. The secondary outcomes were the proportion of babies with BPD (as defined by Jobe and Bancalari 2001^5^), duration of hospitalization, time to regain birth weight, Death, any IVH, IVH Papille grade 3 or 4, PDA requiring medical or surgical therapy, any NEC, NEC modified Bell’s stage – 2 or above^6^, any retinopathy of prematurity (ROP) and ROP requiring treatment as well as the need for a repeat dose of surfactant, the incidence of air leaks and CPAP failure. When the clinical team suspected sepsis based on perinatal risk factors or clinical signs a sepsis screen was performed. The neonate was labelled as having clinical/probable sepsis (clinical and laboratory findings consistent with the bacterial infection without a positive culture) or culture-positive sepsis (presence of the above with a positive blood culture)^7^. Intraventricular haemorrhage^8^ and retinopathy of prematurity^9^ were diagnosed and managed as per the standard guidelines. Echocardiography was done for suspected patent ductus arteriosus and treated if hemodynamically significant^10^.

### Statistical analysis and sample size calculation

Statistical analysis was performed by SPSS software version 23.0. Variables of interest were expressed as percentages, as means (standard deviations) for normally distributed continuous variables, and as medians (ranges) for non-normally distributed variables according to the Kolmogorov–Smirnov test. Fisher’s exact test (two-tailed) or the Mann–Whitney’s U test was used to establish baseline differences between the infants in the LISA and control groups. A *p-*value of < 0.05 was considered statistically significant. The calculated sample size was 75 in each group at a 95% confidence level and 80% study power assuming reduction in the duration of total respiratory support with this new technique by 20% based on previous data from our unit (Mean total duration of respiratory support = 144 h. SD = 60 h).

### Ethics approval

The ethical approval for the study was obtained from the Ethics committee of Human Research Lady Hardinge Medical College, New Delhi, India (LHMC/ECHR/2018/72, dated 14/12/2018).

### Informed consent to participate

Informed consent was obtained from the legal guardian of all included participants.

## Results

A total of 150 infants were enrolled for the study in which 74 neonates were randomized to the LISA group, while 76 infants were randomized to the InSurE group for surfactant administration. Baseline characteristics of subjects in both groups were comparable as depicted in Table 1. The mean birth weight was 1331.47 ± 335.75 gm and the mean gestation age of babies was 30.27 ± 2.07 weeks. Nearly 37% of the mothers received full ANS coverage and 36% of mothers received one or more doses but were not fully covered while 27% of the mothers did not receive any dose of ANS. A high proportion of babies (57%) were delivered by cesarean section. The Median age of surfactant administration was 2 h in both groups but with a statistically significant intra-pharyngeal reflux in babies who received surfactant using the LISA method in comparison to the InSurE method (32% v/s 3%, p < 0.001).

**Table 1:** Baseline characteristics of babies enrolled in study.

Baseline character	LISA (n = 74)	InSurE (n = 76)	P-value
Birth weight (gram) mean, SD	1368 ± 341	1294 ± 328	0.717
Range	660–2200	756–2030	
Gestation age mean, SD	30.48 ± 2.12	30.06 ± 2.01	0.273
Range	26–34	26–34	
26 + 0–27 + 6	05	08	
28 + 0–30 + 6	35	29	
31 + 0–34	34	39	
Male n (%)	45 (60.8%)	42 (55.2%)	0.512
Small for gestation age n (%)	12 (16.2%)	8 (10.5%)	0.305
ANS coverage (complete course) n (%)	28 (37.8%)	28 (36.8%)	0.557
ANS coverage (any dose) n (%)	29 (39.1%)	25 (32.8%)	
No ANS coverage	17 (22.9%)	23 (30.2%)	
Multigravidae n (%)	49 (66.2%)	51 (67.1%)	0.234
Cesarean section delivery n (%)	45 (60.8%)	42 (55.2%)	0.131
Apgar score 5 minutes (median)	8 (8–9)	8 (8–9)	0.55
Requirement of resuscitation n (%)	18 (24.3%)	22 (28.9%)	0.522
Time from birth to surfactant administration (h) median, IQR	2 (2–2)	2 (2–2)	0.814
IP reflux	24 (32.4%)	3 (3.9%)	0.00001
Desaturation episodes	27 (36.4%)	8 (10.5%)	0.0001

**Figure 1:**
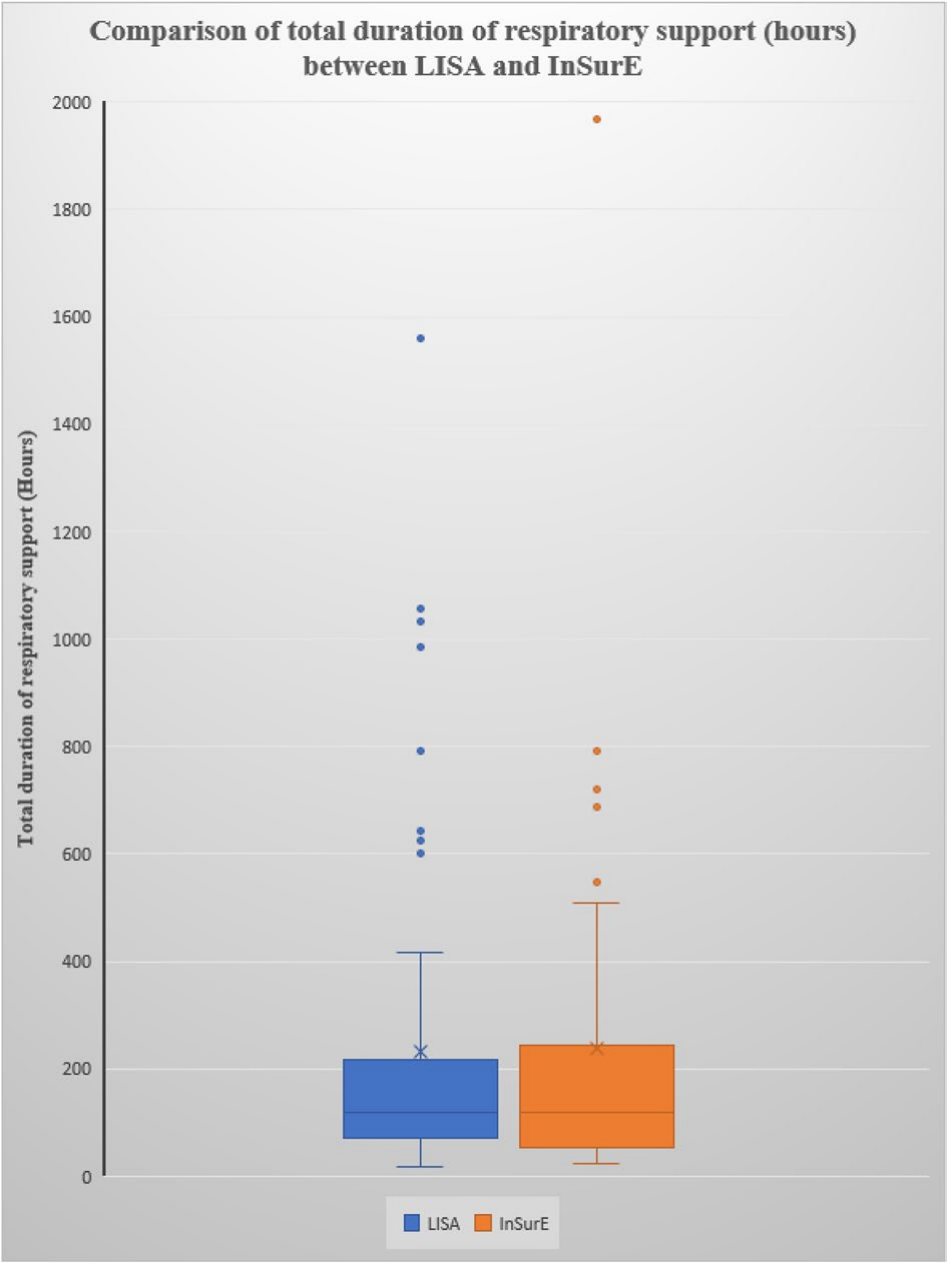
Comparison of the total duration of respiratory support (hours) between Lisa and insure (non-parametric variable, Box-whisker plot).

The results of the primary outcome are depicted as box and whisker plot in Fig. 1 and secondary outcomes are depicted in Table 2. There was no statistically significant difference in the total duration of respiratory support in both groups as depicted in Fig. 1.

**Table 2:** Outcome variables of two groups LISA and InSurE.

Outcome	LISA (n = 74)	InSurE (n = 76)	Total (150)	P value	Relative risk (95% CI)
BPD	6 (8.1%)	6 (7.8%)	12 (8%)	0.962	1.015(0.5622–1.8316)
IVH ≥ II	3 (4.0%)	4 (5.2%)	7 (4.6%)	1	0.863(0.3612–2.0628)
PDA requiring medical or surgical treatment	12 (16.2%)	14 (18.4%)	26 (17.3%)	0.721	0.923 (0.588–1.4491)
NEC stage ≥ II	0 (0%)	1 (1.3%)	1 (0.6%)	1	0.503 (0.04539–5.5814)
Repeat dose of Surfactant	10 (13.5%)	12 (15.7%)	22 (14.6%)	0.694	0.909 (0.5572–1.4831)
Air leaks	1 (1.3%)	0 (0%)	1 (0.6%)	0.493	1.531(0.6764–3.4636)
Need for Invasive Mechanical Ventilation	7 (9.5%)	19 (25%)	26 (17.3%)	0.017	0.498(0.259–0.958)
CPAP failure	13 (17.5%)	29 (38.1%)	42 (28.9%)	0.005	0.548(0.3387–0.8866)
Any sepsis	34 (45.9%)	44 (57.8%)	78 (52%)	0.094	0.554 (0.273–1.121)
ETEF tolerated	50 (84.7%)	52 (89.6%)	102 (87.2%)	0.427	1.224(0.774–1.935)
Death	8 (10.8%)	8 (10.5%)	16 (10.67%)	0.974	1.017(0.365–2.838)

**Figure 2:**
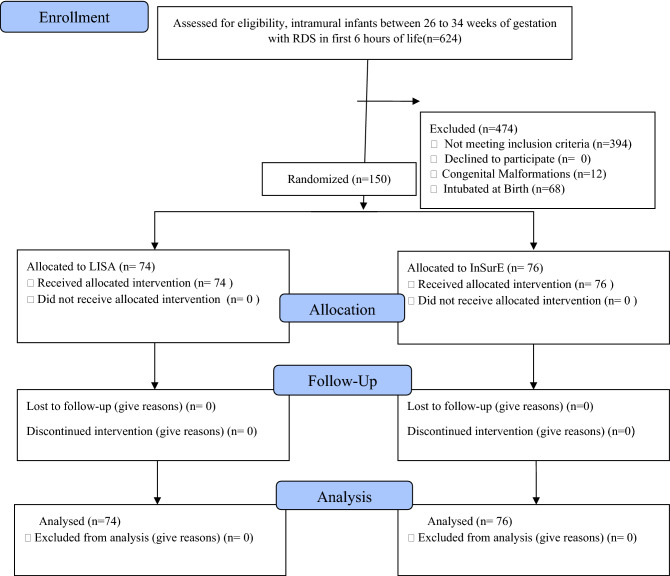
Consort flow chart.

No differences were observed between the two groups for Air leaks, IVH grade II or more, NEC Bell’s stage II or more, PDA requiring medical or surgical management, ROP requiring treatment, Bronchopulmonary dysplasia, total duration of hospital stay, the requirement of the second dose of surfactant, time to regain birth weight and mortality. The need for invasive mechanical ventilation was significantly lower in the LISA group (p = 0.017) with RR (95% CI) 0.498(0.259–0.958). CPAP failure was statistically significantly lower in the LISA group with a p-value of 0.005 and RR (95% CI) 0.548 (0.339–0.887). The median duration of hospital stay was 19 days in LISA group and 26 days in InSurE group with a p-value of 0.207. LISA reduced median duration of hospital stay although the result was not statistically significant (Fig. 2).

## Discussion

In this randomized control trial, we compared LISA with InSurE among 26-to-34-week gestation age infants with RDS in terms of the total duration of respiratory support required. There was no statistically significant difference in the total duration of respiratory support between the two groups. The median duration of total respiratory support was 120 h which was similar in the two groups. The mean duration of total respiratory support was 231.4 ± 292.5 (SD) hours in LISA group and 236.8 ± 359.9 (SD) hours in InSurE group with p-value of 0.92.

In the present study, the need for invasive mechanical ventilatory support was lesser in the LISA group as compared to the InSurE group which was both clinically and statistically significant. Gopel et al. in the AVM trial from Germany and Kanmaz et al. from Turkey also reported a significantly reduced need of mechanical ventilation in the LISA group as compared to InSurE method^11,12^. Jena et al. too in their multi-centre study reported a significantly reduced need for invasive mechanical ventilation^13^. The recently published OPTIMIST-A trial by Dargaville et al. has also reported a significantly reduced need for intubation in the first 72 h in the MIST group^14^.

In the present study we have recorded certain observations during surfactant administration via both the techniques which includes reflux episodes, desaturations, bradycardia and attempts for catheterisation. In this study, Intra-pharyngeal reflux was seen in 32% of babies in the LISA group compared to 3% of babies in the InSurE group. During the procedure, desaturation was observed in 36% of babies in the LISA group compared to 10% of babies in the InSurE group, and the differences were statistically significant. The incidence of bradycardia was 5% in LISA group and 3% in InSurE group which is comparable. In LISA group 20% of the babies required second attempt for catheterisation and due to prior training in pilot phase we were able to administer surfactant via LISA in all the subjects randomized to LISA group.

Kanmaz et al., in their RCT administered porcine surfactant in a dose of 100 mg/Kg as a single bolus over a minute using a 5F feeding tube and reported 21% reflux episodes in the LISA group, which were significantly higher in comparison to the InSurE group (10%) and 18% of babies had desaturation in the LISA group^11^. Heidarzadeh et al. in their study from Iran also reported statistically significant reflux during surfactant administration using thin catheter in comparison to the standard InSurE technique^15^.

In the present study, we used Beractant (Survanta) at a dose of 100 mg/kg (4 ml/kg) over 4 minutes. Curosurf is more concentrated in comparison to Survanta so the required volume for the same dose of surfactant is lesser with Curosurf in comparison to Survanta. Although, majority of studies on LISA have used Curosurf as the surfactant, Kribs et al. used Survanta successfully in their study on the feasibility of surfactant administration in spontaneously breathing extremely premature infants on nCPAP^16^.

In the present study despite the need to use a larger volume of surfactant preparation (Survanta) the numbers of reflux episodes were similar and comparable to the studies from the developed countries in which more concentrated preparation of surfactant (Curosurf) was used requiring smaller volume to achieve the same dose. In the present study, we have used a comparatively wider bore tube as compared to other studies so as to be able to administer the requisite volume of surfactant.

In the present study around approximately 24% of the babies required resuscitation in the form of initial steps in the LISA group and around 29% of the babies in the InSurE group. All the babies enrolled in the study required resuscitation only in the form of initial steps, none of them required bag and mask ventilation which is the reason for median APGAR score of 8 at 5 minutes despite requirement of resuscitation.

As regards the secondary outcome measures in this population of preterm 26–34 weeks of gestation, the incidence of BPD was comparable in the two groups being 8.1% in the LISA group and 7.9% in the InSurE group. The overall incidence of any ROP was very low with no baby requiring laser therapy or surgery for retinopathy of prematurity. In the present study, only one case of NEC stage 2 was reported in the InSurE group and none in the LISA group. The incidence of PDA (20%) was comparable in the two study groups. A recent meta-analysis reported that 35% of babies had PDA in the LISA group with a comparable incidence in InSurE group^17,18^. This low incidence of NEC, PDA, and no baby requiring ROP treatment reflect the adherence of the team to our SOPs for the standard NICU practices which include strict adherence to asepsis routines, early total enteral feeding, increased use of mother’s own milk, and antibiotic stewardship along with DSC and KMC for babies even on respiratory support. The babies enrolled in this study had mean gestation age of 30 weeks with a birth weight of 1300 to 1400 g which also have relatively lesser incidence of BPD and other morbidities described in secondary outcomes as compared to extreme preterms. In our hospital, we provide active care to preterm babies who are 25 weeks of gestation or above and these babies have a comparatively lesser incidence of BPD which is the reason we kept the total duration of respiratory support as our primary outcome.

The babies enrolled in this study had mean gestation age of 30 weeks with a birth weight of 1300 to 1400 g which also have relatively lesser incidence of BPD and other morbidities described in secondary outcomes as compared to extreme preterms. In our hospital, we provide active care to preterm babies who are 25 weeks of gestation or above and these babies have a comparatively lesser incidence of BPD which is the reason we kept the total duration of respiratory support as our primary outcome.

Providing nutrition to preterm babies on respiratory support is always a challenging task. In the present study we had followed early total enteral feeding (ETEF) in hemodynamically stable infants on respiratory support as per our unit policy. We could start early total enteral feed (full volume enteral feed with no intravenous fluid or parenteral nutrition) in 117 babies out of 150 babies from day 1 of life. In thirty-three patients, early total enteral feeding could not be started due to various reasons—7 babies had Absent/reverse end diastolic flow (A/REDF) on antenatal doppler,15 babies had shock and required inotropic support on day 1 of life, 8 babies had high ventilatory settings at the time of admission and in 3 babies, abdomen was not soft to start total enteral feeding. Out of the 117 babies in which ETEF was started, 102 tolerated ETEF, and 15 had one or more episodes of FI requiring parenteral supplementation but none developed NEC. The one baby who developed NEC was not started on ETEF as baby had REDF on antenatal doppler studies.

There was no statistically significant difference in the requirement of the second dose of surfactant between the two groups. In a study by Augur et al., the requirement for the second dose of surfactant was significantly higher in the MIST group in comparison to InSurE group (35.6% vs 6.5%, p = 0.003). This finding could have occurred because the dose of surfactant in the MIST procedure was 100 mg/kg compared with 200 mg/kg in the InSurE group. In the present study, we used the same dose of surfactant in both groups and the findings were consistent with previous trials showing good results with a dose of 100 mg/kg^12,18^.

There was a trend toward reduced requirement of antibiotics in the intervention group although the results were not statistically significant. In this study, the length of hospital stay was also shorter in the LISA group although the results were not statistically significant but the reduction of seven days of hospital stay is clinically quite significant and has a major social and economic impact, especially in the backdrop of LMIC. The early discharge is influenced by several factors which include complications occurring during NICU stay (sepsis, feeding problems), social factors (parental presence and involvement), and public health factors^19^.

Various trials have reported that the incidence of nCPAP failure in the first 7 days of life is 46 to 51% which ultimately increases the need for mechanical ventilation^20–22^. In the present study CPAP failure was also significantly reduced in the LISA group (p = 0.005) with RR (95% CI) 0.55 (0.34–0.89). Most of the studies on the LISA method have not reported this clinically relevant outcome precluding comparison with other studies. Avoidance of mechanical ventilation after surfactant administration is likely to prevent various morbidities postulated to result from biotrauma, volutrauma, and barotrauma^23^.

In the present study, we have enrolled babies between 26 and 34 weeks of gestation age which covers almost the complete spectrum of RDS in lower and middle-income countries. This study has shown that LISA with an oro-gastric feeding tube is feasible as well as effective. An oro-gastric feeding tube is inexpensive and easily available in all neonatal units. The cost of special LISA catheter is approximately 30 USD in India which is 100 times of cost of the feeding tube. The ease of training for using the feeding tube for LISA makes it a promising tool for surfactant administration in lower-middle-income countries like ours.

This study is a single-center study as it was part of a time-bound dissertation We did not use any premedication for sedation before LISA or InSurE and used only non-pharmacological methods like nesting and swaddling for the baby’s comfort. Moschino et al. in their recently published systematic review have shown that the use of sedative drugs for LISA in preterm neonates with RDS might be associated with an increased risk of desaturation and requirement of nIPPV^24^. The lack of sedation for both interventions may present ethical problems in some countries. During InSurE technique, we have used bag & tube ventilation between aliquots of surfactant rather than pressure limited volume guarantee ventilation using a mechanical ventilator. This may introduce a bias for probably increased lung injury^25^. There was no blinding of the study physicians in the application of the study procedure nor were the outcome assessors blinded.

The present study concludes that LISA with an 8F feeding tube is a feasible and effective strategy for surfactant administration which resulted in a significant reduction in the need for invasive mechanical ventilatory support. There are certain unresolved questions with the LISA method that are related to the ideal type of catheters, the need for premedication or sedation, the most suited surfactant preparation, and the duration of surfactant administration during LISA. Multicentric trials would be required to address these questions.

## Data Availability

The data that support the findings of this study are available from the corresponding author, upon reasonable request.

## Abbreviations

ANS: Antenatal corticosteroids
BPD: Bronchopulmonary dysplasia
CI: Confidence interval
FiO2: Fraction of oxygen in inspired air
hs PDA: Hemodynamically significant patent ductus arteriosus
IQR: Interquartile range
InSurE: Intubate surfactant extubate
IVH: Intraventricular hemorrhage
LISA: Less invasive surfactant administration
nCPAP: Nasal continuous positive airway pressure
NIPPV: Non-invasive positive pressure ventilation
NICU: Neonatal intensive care unit
PEEP: Positive end-expiratory pressure
PIP: Peak inspiratory pressure
RDS: Respiratory distress Syndrome
RR: Relative risk

## References

[1] Kattwinkel J, Robinson M, Bloom BT, Delmore P, Ferguson JE (2004). Technique for intrapartum administration of surfactant without requirement for an endotracheal tube. J. Perinatol..

[2] Isayama T, Iwami H, McDonald S, Beyene J (2016). Association of noninvasive ventilation strategies with mortality and bronchopulmonary dysplasia among preterm infants: A systematic review and meta-analysis. JAMA.

[3] Shim GH (2017). Update of minimally invasive surfactant therapy. Korean J. Pediatr..

[4] Verder H, Robertson B, Greisen G, Ebbesen F, Albertsen P, Lundstrom K, Jacobsen T (1994). Surfactant therapy and nasal continuous positive airway pressure for newborns with respiratory distress syndrome. N. Engl. J. Med..

[5] Jobe AH, Bancalari E (2001). Bronchopulmonary dysplasia: NICHD/NHLBI/ORD workshop summary. Am. J. Respir. Crit. Care Med..

[6] Walsh MC, Kliegman RM (1986). Necrotizing enterocolitis: Treatment based on staging criteria. Pediatr. Clin. N. Am..

[7] Mittal K, Gupta V, Khanna P, Kaushik JS, Sharma A (2014). Evaluation of integrated management of neonatal and childhood illness (IMNCI) algorithm for diagnosis and referral in under-five children. Indian J. Pediatr..

[8] Papile LA, Burstein J, Burstein R, Koffler H (1978). Incidence and evolution of subependymal and intraventricular hemorrhage: A study of infants with birth weights less than 1,500 gm. J. Pediatr..

[9] International Committee for the Classification of Retinopathy of Prematurity (2005). International classification of retinopathy of prematurity revisited. Arch. Ophthalmol..

[10] Skelton R, Evans N, Smythe J (1994). A blinded comparison of clinical and echocardiographic evaluation of the preterm infant for patent ductus arteriosus. J. Paediatr. Child. Health..

[11] Göpel W, Kribs A, Ziegler A, Laux R, Hoehn T, Wieg C, Siegel J, Avenarius S, von der Wense A, Vochem M, Groneck P (2011). Avoidance of mechanical ventilation by surfactant treatment of spontaneously breathing preterm infants (AMV): An open-label, randomized, controlled trial. The Lancet..

[12] Kanmaz HG, Erdeve O, Canpolat FE, Mutlu B, Dilmen U (2013). Surfactant administration via thin catheter during spontaneous breathing: Randomized controlled trial. Pediatrics.

[13] Jena SR, Bains HS, Pandita A, Verma A, Gupta V, Kallem VR, Abdullah M, Kawdiya A (2019). Surfactant therapy in premature babies: SurE or InSurE. Pediatr. Pulmonol..

[14] Dargaville PA, Kamlin CO, Orsini F, Wang X, De Paoli AG, Kutman HG (2021). Effect of minimally invasive surfactant therapy vs sham treatment on death or bronchopulmonary dysplasia in preterm infants with respiratory distress syndrome: The OPTIMIST-A randomized clinical trial. JAMA.

[15] Mohammadizadeh M, Ardestani AG, Sadeghnia AR (2015). Early administration of surfactant via a thin intratracheal catheter in preterm infants with respiratory distress syndrome: Feasibility and outcome. J. Res. Pharm. Pract..

[16] Kribs A, Pillekamp F, Huenseler C, Vierzig A, Roth B (2007). Early administration of surfactant in spontaneous breathing with nCPAP: Feasibility and outcome in extremely premature infants (postmenstrual age ≤ 27 weeks). Pediatr. Anesth..

[17] Aldana-Aguirre JC, Pinto M, Featherstone RM, Kumar M (2017). Less invasive surfactant administration versus intubation for surfactant delivery in preterm infants with respiratory distress syndrome: A systematic review and meta-analysis. Arch. Dis. Child. Fetal Neonatal Ed..

[18] Lau CS, Chamberlain RS, Sun S (2017). Less invasive surfactant administration reduces the need for mechanical ventilation in preterm infants: A meta-analysis. Glob. Pediatr. Health..

[19] Aguar M, Cernada M, Brugada M, Gimeno A, Gutierrez A, Vento M (2014). Minimally invasive surfactant therapy with a gastric tube is as effective as the intubation, surfactant, and extubation technique in preterm babies. Acta Paediatr..

[20] Gupta BK, Saha AK, Mukherjee S, Saha B (2020). Minimally invasive surfactant therapy versus InSurE in preterm neonates of 28 to 34 weeks with respiratory distress syndrome on non-invasive positive pressure ventilation: A randomized controlled trial. Eur. J. Pediatr..

[21] Morley CJ, Davis PG, Doyle LW, Brion LP, Hascoet JM, Carlin JB (2008). Nasal CPAP or intubation at birth for very preterm infants. N. Engl. J. Med..

[22] Finer NN, Carlo WA, Walsh MC (2010). Early CPAP versus surfactant in extremely preterm infants. N. Engl. J. Med..

[23] Dunn MS, Kaempf J, de Klerk A (2011). Randomised trial comparing 3 approaches to the initial respiratory management of preterm neonates. Pediatrics.

[24] Moschino L, Ramaswamy VV, Reiss IK, Baraldi E, Roehr CC, Simons SH (2022). Sedation for less invasive surfactant administration in preterm infants: A systematic review and meta-analysis. Pediatr. Res..

[25] De Luca D, Aguilera SS, Centorrino R, Fortas F, Yousef N, Carnielli VP (2020). Less invasive surfactant administration: A word of caution. Lancet Child Adolesc. Health.

